# Network toxicology and bioinformatics analysis predict potential molecular targets and mechanisms by which sevoflurane and propofol influence type 2 diabetes mellitus

**DOI:** 10.1371/journal.pone.0349565

**Published:** 2026-05-18

**Authors:** Lili Bai, Ping Li, Lina Zhao

**Affiliations:** Department of Anesthesia, Tianjin Hospital, Tianjin, China; Sapienza University of Rome: Universita degli Studi di Roma La Sapienza, ITALY

## Abstract

**Background:**

Sevoflurane and propofol are commonly used anesthetics that may exert pronounced effects on glucose metabolism and cardiovascular function in patients with type 2 diabetes mellitus (T2DM). This study aimed to elucidate the molecular mechanisms underlying the association of sevoflurane and propofol with T2DM progression.

**Methods:**

Targets associated with sevoflurane, propofol, and T2DM were predicted using public databases. Core targets were identified and validated through differential expression analysis, machine learning algorithms, and expression level verification. Functional enrichment analysis was conducted to characterize related biological processes. Immune cell infiltration was assessed using the CIBERSORT algorithm, and regulatory networks involving microRNAs and transcription factors were constructed. Molecular docking was performed to evaluate the binding affinities between the anesthetics and the core targets.

**Results:**

Two core targets, MMP9 (Matrix Metallopeptidase 9) and HPSE (Heparanase), were identified as significantly associated with sevoflurane and propofol in T2DM. Supplementary sensitivity analyses across multiple fold-change thresholds and permutation tests demonstrated that MMP9 and HPSE were retained as candidate targets across a range of analytical choices, supporting the robustness of their identification. These targets were enriched in pathways related to receptor catabolic processes, cellular responses to abiotic stimuli, and autophagy. Immune infiltration analysis indicated increased neutrophil abundance and decreased levels of activated natural killer (NK) cells. In addition, 16 microRNAs and 12 transcription factors were associated with the core targets. Molecular docking demonstrated potential interactions of sevoflurane and propofol with MMP9 and HPSE.

**Conclusion:**

These findings provide novel insights into the potential association between exposure to sevoflurane and propofol and the progression of T2DM, and identify candidate therapeutic targets. Further experimental and clinical validation is required to support evidence-based selection of personalized anesthetic strategies for patients with T2DM.

## 1. Introduction

Diabetes mellitus (DM) is a metabolic disorder characterized by chronic hyperglycemia and has become a major global public health challenge. According to the International Diabetes Federation (IDF) Diabetes Atlas (2021), the global prevalence of diabetes was 10.5% in 2021 and is projected to reach 12.2% by 2045 [1]. Its development is primarily associated with insulin resistance (IR) and/or impaired insulin secretion. Clinically, DM is classified into type 1 diabetes mellitus (T1DM), type 2 diabetes mellitus (T2DM), gestational diabetes, and other specific types [2]. Among these, the prevalence of T2DM continues to increase. Global data indicate that the prevalence of T2DM was 9.3% in 2019 and is projected to reach 10.9% by 2045 [3]. As a chronic metabolic disease, T2DM is characterized by key pathological features including IR, β-cell dysfunction, and insufficient compensatory insulin secretion in response to hyperglycemia [4]. In addition, patients with T2DM face a substantially increased risk of mortality and cardiovascular diseases [5]. With the expanding role of anesthesiology in perioperative medicine, increasing attention has been directed toward the impact of anesthetic management on surgical outcomes. In patients with T2DM, acute severe hypoglycemia during the induction of general anesthesia, although uncommon, represents a potentially life-threatening complication. Such episodes may result in neurological dysfunction, cognitive impairment, and even irreversible brain injury [6], and may also precipitate cardiovascular events, including arrhythmias and myocardial ischemia [7–9].

Maintenance of general anesthesia is typically achieved using intravenous agents (e.g., propofol) or inhalational anesthetics (e.g., sevoflurane) [10]. Propofol is a potent intravenous anesthetic that exerts its effects by enhancing γ-aminobutyric acid (GABA)-mediated inhibitory neurotransmission at GABA receptors [11,12]. Patients with T2DM are frequently older and physically vulnerable, often presenting with organ dysfunction, immune impairment, and heightened stress responses, all of which reduce perioperative tolerance to anesthetic agents and increase the risk of intraoperative complications. Importantly, propofol and its lipid emulsion carrier may induce or aggravate IR in the diabetic myocardium by disrupting insulin signaling pathways [13]. Sevoflurane is a commonly used volatile anesthetic, favored for its low blood-gas solubility, rapid induction and recovery, and ease of anesthetic depth control during maintenance [14]. Experimental studies have shown that sevoflurane can aggravate hippocampal neuroinflammation, leading to acute cognitive impairment and persistent cognitive dysfunction [15]. Volatile anesthetics may also suppress insulin secretion through activation of ATP-sensitive potassium (KATP) channels in pancreatic β-cells, impair insulin signaling, and rapidly induce hepatic IR [16], thereby increasing the risk of severe hyperglycemia compared to intravenous anesthesia [17]. Sevoflurane-induced QTc prolongation has been reported to be more pronounced in patients with chronic hyperglycemia than in normoglycemic individuals [18]. In addition, sevoflurane anesthesia is associated with higher postoperative blood glucose levels and greater increases in inflammatory mediators, including IL-6 and TNF-α, compared to propofol [19]. Patients receiving total inhalational anesthesia exhibit higher early postoperative blood glucose and cortisol levels, whereas those undergoing total intravenous anesthesia show higher postoperative insulin levels [17]. Despite these findings, the specific molecular targets and signaling pathways linking sevoflurane and propofol to T2DM remain unclear, which limits the development of effective intervention strategies targeting anesthetic-related metabolic toxicity.

Network toxicology has emerged as an interdisciplinary approach that integrates bioinformatics, systems biology, and toxicology to systematically characterize the molecular interactions between chemicals and biological systems and to elucidate their mechanisms of action [20,21]. This approach offers significant advantages for toxicity research by enabling efficient target identification and providing a novel framework for analyzing complex toxicological mechanisms. T2DM is a multifactorial systemic metabolic disorder characterized not only by metabolic dysfunction in classical target organs but also by a state of chronic low-grade systemic inflammation [22]. These inflammatory and metabolic alterations are reflected in the circulatory system (e.g., peripheral blood). Transcriptomic profiles of immune cells, such as peripheral blood mononuclear cells (PBMCs), have been shown to mirror disease progression and severity in T2DM [23]. Accordingly, analysis of peripheral blood transcriptomic data enables systematic identification of key regulatory networks associated with T2DM. Based on this systemic perspective, the present study aimed to identify core targets related to anesthetic-associated toxicity and to generate mechanistic hypotheses regarding their downstream effects on specific metabolic organs.

## 2. Materials and methods

### 2.1. Collection of type 2 diabetes mellitus-related data

Transcriptomic data from RNA sequencing (RNA-seq) datasets associated with T2DM (GSE21321 and GSE95849) were obtained from the Gene Expression Omnibus (GEO) database (accessed July 5, 2025; http://www.ncbi.nlm.nih.gov/geo/). For GSE21321 [24] (platform: GPL6883, training set), nine peripheral blood samples from patients with T2DM and eight samples from healthy controls were included. For GSE95849 [25] (platform: GPL22448, validation set), six peripheral blood samples from patients with T2DM and six from normal controls were selected. In addition, the keyword “T2DM” was used to retrieve disease-associated targets from the GeneCards database (accessed July 5, 2025; https://www.genecards.org/). The resulting entries were imported into the Universal Protein Resource (UniProt) database (https://www.uniprot.org/) to obtain standardized gene symbols. After removing entries lacking gene annotations, a total of 1,617 T2DM-related targets were identified (S1 Table).

### 2.2. Toxicity analysis of sevoflurane and propofol and acquisition of related targets

As a preliminary step in the network toxicology analysis, the absorption, distribution, metabolism, excretion, and toxicity (ADMET) profiles of sevoflurane and propofol were evaluated using ADMETlab 3.0 (https://admetlab3.scbdd.com/server/screening). The SMILES structures of sevoflurane (C(OC(C(F)(F)F)C(F)(F)F)F) and propofol (CC(C)C1 = C(C(=CC = C1)C(C)C)O) were obtained from the PubChem database (https://pubchem.ncbi.nlm.nih.gov/). Potential toxicity-related targets of sevoflurane and propofol were predicted using the Comparative Toxicogenomics Database (CTD) (accessed July 9, 2025; https://ctdbase.org/) and the PharmMapper database (accessed July 9, 2025; https://lilab-ecust.cn/pharmmapper/). Target names were standardized using the UniProt database, and the organism was restricted to “Homo sapiens”. After removal of duplicate entries, 1,040 sevoflurane- and propofol-associated targets were retained (S2 Table). As a supplementary sensitivity analysis to enhance biological relevance, we applied context-aware filtering: CTD targets were restricted to those annotated as “direct interaction” and species “Homo sapiens”. PharmMapper targets were limited to the top 20% of Fit scores. Both target sets were further filtered to genes with detectable expression (count > 0 in at least one sample) in the GSE21321 or GSE95849 datasets.

### 2.3. Candidate targets acquisition

Differential expression analysis between T2DM and control samples in GSE21321 was performed using the limma package (v 3.19) [26], with thresholds of |log_2_ fold change (FC)| > 0.5 and p < 0.05 to identify differentially expressed genes (DEGs). The expression patterns of DEGs were visualized using the ggplot2 package (v 3.4.2) and the ComplexHeatmap package (v 2.12.1) [27]. To address concerns about multiple testing control, we supplemented the initial DEG identification with an analysis applying Benjamini-Hochberg false discovery rate (FDR) correction (adjusted p < 0.05, |log_2_FC| > 0.5). Candidate targets were identified by intersecting T2DM-related targets, sevoflurane- and propofol-related targets, and DEGs using the ggvenn package (v 0.1.9) [28]. Using the corresponding platform gene sets as background, statistical significance of the overlap was evaluated by a hypergeometric test implemented with the phyper() function in the stats package (v 4.2.2, https://www.r-project.org/). We also conducted permutation tests (10,000 iterations) to evaluate whether the observed overlaps between DEGs, anesthetic targets, and T2DM targets are statistically significant compared to random expectation.

### 2.4. Functional enrichment analysis and protein-protein interaction network construction

To characterize the biological functions of candidate targets in T2DM, Gene Ontology (GO) and Kyoto Encyclopedia of Genes and Genomes (KEGG) enrichment analyses were conducted using the clusterProfiler package (v 4.10.0) [29], with an adjusted p value < 0.05. Candidate targets were then imported into the Search Tool for the Retrieval of Interacting Genes/Proteins (STRING) database (https://cn.string-db.org/) to construct a protein-protein interaction (PPI) network with an interaction score > 0.4, followed by visualization and analysis in Cytoscape software (v 3.10.2) [30]. Protein importance within the network was evaluated using the CentiScaPe 2.2 and ClueGO plugins in Cytoscape, which calculated multiple centrality measures, including degree, betweenness, and stress. PPI nodes were ranked based on mean normalized scores, and proteins with higher composite centrality values were prioritized.

### 2.5. Machine learning algorithms and expression level verification

Across all samples in the GSE21321 dataset, three machine learning algorithms—least absolute shrinkage and selection operator (LASSO), Extreme Gradient Boosting (XGBoost), and Boruta—were applied to identify signature targets associated with T2DM from the candidate target set. The glmnet package (v 4.1) [31] was used to determine LASSO-derived signature targets, with the optimal lambda selected through five-fold cross-validation. To address the limitations of small sample size (n = 17), we implemented nested cross-validation (5-fold outer loop × 5-fold inner loop) with 100 repetitions. Bootstrap resampling (1,000 iterations) was used to estimate 95% confidence intervals for performance metrics. This approach provides strong generalization performance and is well suited for high-dimensional transcriptomic data with multicollinearity. XGBoost, a widely used supervised ensemble learning method, was implemented using the XGBoost package (v 1.7.5.1) [32] to construct a classification model and identify key feature signatures, followed by feature importance-based selection of XGBoost-derived targets. Feature importance was further evaluated using the Boruta algorithm implemented in the Boruta package (v 7.0.0) [33]. By comparing the importance of each candidate feature with that of randomly generated shadow features, Boruta-derived signature targets were identified. The intersection of LASSO-, XGBoost-, and Boruta-derived targets was determined using the ggvenn package (v 0.1.9) to obtain key signature targets.

The expression levels of key signature targets were subsequently evaluated in both the GSE21321 and GSE95849 datasets using the Wilcoxon test (p < 0.05). Targets showing significant differences and consistent expression trends between T2DM and control groups across both datasets were defined as core targets.

### 2.6. Supplementary analyses for robustness validation

To validate the robustness of the identified core targets, we performed several supplementary analyses. These included: (1) sensitivity analyses across six fold-change thresholds (|log₂FC| > 0.25, 0.50, 0.75, 1.00, 1.25, 1.50) to assess whether core target retention depends on threshold selection; (2) context-aware filtering to assess target retention under more stringent conditions: CTD targets were restricted to those annotated as “direct interaction” and species “Homo sapiens”. PharmMapper targets were limited to the top 20% of Fit scores. Both target sets were further filtered to genes with detectable expression (count > 0 in at least one sample) in the GSE21321 or GSE95849 datasets); and (3) drug-specific parallel analyses for sevoflurane and propofol to assess whether core targets are consistently prioritized for both anesthetics.

### 2.7. Gene multiple association network integration algorithm and gene set enrichment analysis

A co-expression network of the core targets and functionally related genes was constructed using the GeneMANIA database (http://genemania.org/).

Gene set enrichment analysis (GSEA) was performed using the clusterProfiler package (v 4.10.0) to investigate biological functions and signaling pathways associated with the core targets (p < 0.05, false discovery rate [FDR] < 0.25, |normalized enrichment score [NES]| > 1). For all samples in GSE21321, Spearman correlation coefficients between each core target and all other genes were calculated using the psych package (v 2.3.6) [34]. Genes were ranked in descending order according to correlation values to generate target-specific gene lists. The background gene set (c5.bp.v2022.1.Hs.symbols.gmt) was obtained from the Molecular Signatures Database (MSigDB) (https://www.gsea-msigdb.org/gsea/msigdb/).

### 2.8. Immune microenvironment analysis

Significant alterations in immune cell composition may reflect immune regulatory imbalance in T2DM and have clinical and pathological relevance for disease assessment and therapeutic evaluation [35]. Based on the GSE21321 dataset, immune cell infiltration was estimated using the CIBERSORT algorithm to quantify the relative abundance of 22 immune cell types [36]. Samples with CIBERSORT p-values > 0.05 were excluded. Differences in immune cell infiltration between patients with T2DM and healthy controls were subsequently evaluated using the Wilcoxon rank-sum test, with statistical significance defined as p < 0.05. To address multiple comparison concerns, we additionally applied the Benjamini-Hochberg FDR correction as a supplementary analysis.

### 2.9. Molecular regulatory network

MicroRNAs (miRNAs) play essential roles in biological development and evolution by regulating key target genes. To clarify the molecular regulatory mechanisms of core targets in T2DM, miRNAs targeting these genes were identified using the StarBase database (https://starbase.sysu.edu.cn/). Candidate miRNAs were screened using the threshold “pancancerNum > 6” for subsequent analysis. Transcription factors (TFs), as critical regulators, control gene expression at the transcriptional level through binding to target sequences. Relevant TFs were identified by querying the ChIPBase database (http://rna.sysu.edu.cn/chipbase/), with selection criteria requiring the combined number of upstream and downstream detected samples to exceed 12. Regulatory networks illustrating miRNA-mRNA and TF-mRNA interactions were subsequently constructed using the Cytoscape platform (version 3.10.2).

### 2.10. Molecular docking

Molecular docking analysis was conducted to evaluate the binding affinities between compounds (sevoflurane and propofol) and core targets. Three-dimensional (3D) structures of sevoflurane, propofol, and the positive control drug were obtained from the PubChem database, while the 3D structures of core targets were retrieved from the PDB database (https://www.rcsb.org/). Following dehydration, hydrogen addition, and charge neutralization, the CB-Dock2 platform (https://cadd.labshare.cn/cb-dock2/index.php) was used to predict binding sites and affinities of protein-ligand complexes. Docking results were visualized using PyMOL (v 2.4.0) [37], and binding energies ≤ −5.0 kcal/mol were considered indicative of favorable interactions.

### 2.11. Statistical analysis

Statistical analyses were performed in the R environment (version 4.4.0) for data processing and computation. Differences between groups were assessed using the Wilcoxon rank-sum test, with statistical significance defined as p < 0.05.

## 3. Results

### 3.1. Chemical structures and toxicity identifications of sevoflurane and propofol

The 2D molecular structures of sevoflurane and propofol are presented in S1a-b Fig. Toxicological profiles were evaluated using the ADMETlab 3.0 platform, which provides probability scores (0–1) for multiple toxicity endpoints (e.g., hepatotoxicity and skin sensitization), with higher values indicating greater toxicity risk. The predictions suggested that sevoflurane carries a higher risk of human hepatotoxicity (0.778), whereas propofol shows a greater likelihood of inducing skin sensitization (0.702). These results indicate distinct toxicity profiles and biological effect potentials for sevoflurane and propofol (S3 Table).

### 3.2. Functional evaluation of candidate targets and identification of protein-protein interaction targets

**Fig 1:**
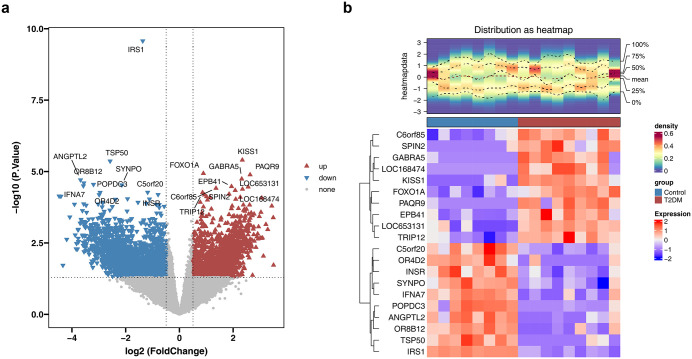
Differentially expressed genes (DEGs) between type 2 diabetes mellitus (T2DM) and control samples. **(a)** Volcano plot of DEGs (|log_2_FoldChange| > 0.5, **P.**Value < 0.05). **(b)** Heatmap of DEGs.

**Fig 2:**
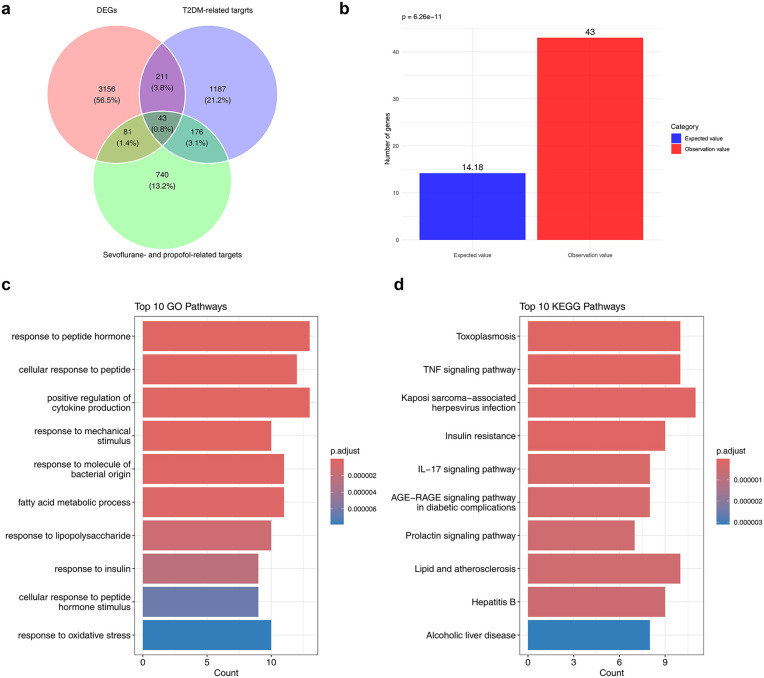
Identification and functional enrichment of candidate targets. **(a)** Venn diagram showing the intersection of candidate targets. **(b)** Hypergeometric distribution analysis of candidate genes. **(c)** Gene ontology (GO) enrichment of candidate targets (adj p < 0.05). **(d)** Kyoto encyclopedia of genes and genomes (KEGG) pathway enrichment of candidate targets (adj p < 0.05).

In the GSE21321 dataset, 3,491 DEGs (S4 Table) were identified between the T2DM and control groups (|log_2_ FC| > 0.5, p < 0.05), including 1,681 upregulated and 1,810 downregulated genes in T2DM. A volcano plot and heatmap were constructed to display the top 10 most significantly regulated (up/down) DEGs based on the smallest p values and their differential expression patterns between groups (Fig 1a-b). To address multiple testing concerns, we performed a supplementary analysis using Benjamini-Hochberg FDR correction (adjusted p < 0.05, |log₂FC| > 0.5), which yielded only 13 DEGs. This overly stringent threshold would severely limit biological interpretability and was not optimal for exploratory network toxicology studies. Therefore, we retained the original uncorrected results (3,491 DEGs) for subsequent analyses to maximize sensitivity for target discovery. The FDR-corrected results were provided as a supplementary reference (S2a-b Fig). A total of 1,040 sevoflurane- and propofol-associated targets were systematically retrieved from the CTD and PharmMapper databases, representing potential molecular mediators of their toxicological effects. The overlap among 1,040 anesthetic-related targets, 1,617 T2DM-associated targets, and 3,491 DEGs yielded 43 candidate targets (Fig 2a, S5 Table). Hypergeometric testing demonstrated that this overlap was unlikely to occur by chance (p = 6.62e-11) (Fig 2b), indicating a statistically significant enrichment and supporting the non-random selection of these candidates. Permutation testing (10,000 iterations) at FC = 0.50 showed that the observed overlap was not statistically distinguishable from random expectation (overlap = 43, p = 0.244, fold enrichment = 1.1×) (S3a-b Fig). Across all FC thresholds, fold enrichment ranged from 0.8× to 1.1 × , none reaching statistical significance (S3c Fig). Given that permutation testing is more conservative and does not assume gene independence, the overlap should be interpreted with caution. We therefore consider these findings hypothesis-generating and in need of experimental validation. GO and KEGG enrichment analyses were subsequently performed to characterize their functional relevance. GO analysis identified significant enrichment in 1,001 biological processes, including “response to peptide hormone” and “fatty acid metabolic process” (adj p < 0.05, Fig 2c, S6 Table). KEGG analysis further revealed that 37 candidate targets were involved in 87 signaling pathways, notably the “TNF signaling pathway” and “IL-17 signaling pathway” (adj p < 0.05, Fig 2d, S7 Table).

**Fig 3:**
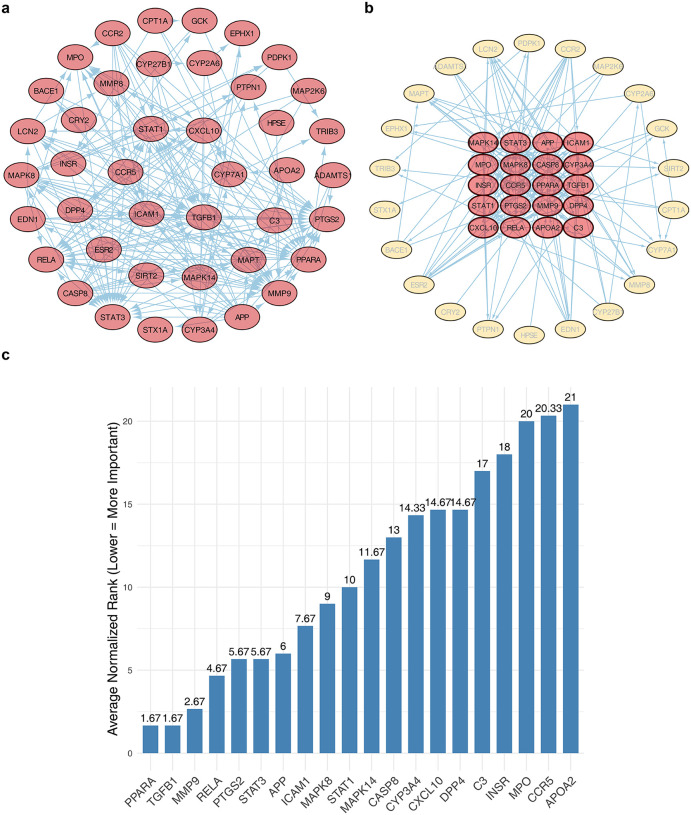
Construction of the protein-protein interaction (PPI) network and identification of topologically central nodes among candidate targets. **(a)** PPI network of candidate targets (interaction score > 0.4). Each ellipse represents a target. **(b)** Network of key PPI targets displayed in a radial layout, with the inner circle containing the top 20 ranked genes. **(c)** Top 20 genes ranked by average normalized centrality score; higher-ranked genes exhibit greater network centrality.

Protein-protein interactions among the 43 candidate targets were analyzed, resulting in a PPI network containing 42 proteins and 202 interaction pairs. Within this network, STAT1, CXCL10, CYP7A1, TGFB1, ICAM1, and CCR5 displayed extensive connectivity with other proteins (Fig 3a). Topological analysis using the CentiScaPe 2.2 and ClueGO plugins identified 20 key PPI targets: PPARA, TGFB1, MMP9, RELA, PTGS2, STAT3, APP, ICAM1, MAPK8, STAT1, MAPK14, CASP8, CYP3A4, CXCL10, DPP4, C3, INSR, MPO, CCR5, and APOA2 (Fig 3b-c). The functional relevance of these targets under anesthetic conditions, however, remains to be established and requires validation through condition-specific interactomic analyses and targeted gene perturbation experiments.

### 3.3. Identification of matrix metallopeptidase 9 (MMP9) and heparanase (HPSE) as core targets for type 2 diabetes mellitus

**Fig 4:**
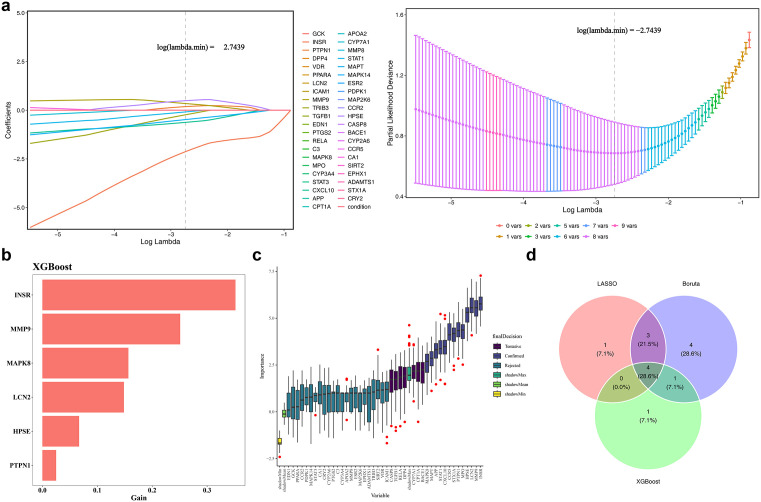
Identification of core T2DM targets and model construction using machine learning algorithms. **(a)** Signature target selection using the least absolute shrinkage and selection operator (LASSO) algorithm (left: coefficient profiles; right: five-fold cross-validation error curve; log(lambda.min) = −2.7439). **(b)** Feature importance ranking of signature targets identified by the XGBoost algorithm, highlighting genes with the greatest contribution to T2DM classification. **(c)** Box plot of signature targets identified by the Boruta algorithm. Default parameters were applied (maxRuns = 1000), and variable importance was evaluated using shadow feature comparison. **(d)** Venn diagram showing the overlap of signature targets identified by the three machine learning methods.

Machine learning approaches were applied to identify signature targets associated with T2DM. The LASSO model selected eight signature targets (INSR, MMP9, MAPT, CXCL10, STAT1, CYP7A1, TRIB3, and CA1) at the optimal penalty parameter (log(lambda.min) = −2.7439; Fig 4a). Using nested cross-validation (5 × 5, 100 repetitions) and bootstrap resampling (1,000 iterations), the LASSO model selected signature targets at the optimal penalty parameter. The nested cross-validation yielded a mean AUC of 0.942 (95% CI: 0.931–0.953), with accuracy of 0.813 (95% CI: 0.802–0.824) (S4a-b Fig). Bootstrap OOB evaluation (1,000 iterations) gave a mean AUC of 0.911 (95% CI: 0.903–0.920) and accuracy of 0.778 (95% CI: 0.767–0.789) (S4c-d Fig). The frequency with which MMP9 and HPSE were selected across all bootstrap iterations was 25.7% and 35.4%, respectively (S4e Fig). The XGBoost model identified six signature targets, including INSR, MMP9, MAPK8, LCN2, HPSE, and PTPN1, based on feature importance rankings (Fig 4b). The Boruta algorithm further confirmed 12 relevant targets for model selection: INSR, HPSE, MMP9, MAPT, CXCL10, CCR5, APP, LCN2, PTPN1, MPO, STAT1, and STX1A (Fig 4c). Intersection of the results from the three algorithms yielded four key signature targets: INSR, PTPN1, MMP9, and HPSE (Fig 4d).

**Fig 5:**
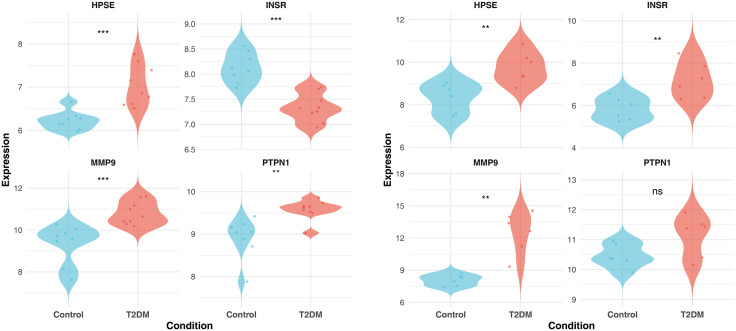
Violin plots showing expression validation of key targets in T2DM and control groups (left: training set; right: validation set) (Wilcoxon test, **p < 0.01, ***p < 0.001, ns indicates no significant difference).

Expression patterns of these key targets were subsequently validated in T2DM and control samples from the GSE21321 and GSE95849 datasets. MMP9 and HPSE showed significantly increased expression in the T2DM group (p < 0.01, Fig 5).

### 3.4. Supplementary validation of core target robustness

After identifying MMP9 and HPSE as core targets through the primary analytical pipeline, we conducted several supplementary analyses to validate the robustness of these findings. To assess whether the identification of MMP9 and HPSE depends on the choice of fold-change threshold, we performed sensitivity analyses across six thresholds (|log₂FC| > 0.25, 0.50, 0.75, 1.00, 1.25, 1.50). The number of DEGs decreased from 4,034 at FC = 0.25 to 1,811 at FC = 1.50, and candidate targets decreased from 47 to 15 (S5a-b Fig). Both MMP9 and HPSE were retained at FC = 0.25–0.75; at least one was retained at FC = 1.00–1.25; and both were lost only at FC = 1.50, indicating robust identification under moderate threshold selections (S5c Fig).

To test whether MMP9 and HPSE remain identified under more stringent target selection criteria, we applied context-aware filtering: CTD targets were restricted to those annotated as “direct interaction” and species “Homo sapiens”. PharmMapper targets were limited to the top 20% of Fit scores (S6a-b Fig). Both target sets were further filtered to genes with detectable expression. Under these stricter conditions, the candidate target set was reduced from 43 to 33, and MMP9 and HPSE were retained (S6c Fig, S8 Table). Hypergeometric testing with the refined background gave p = 7.77e-14 (GSE21321) and p = 4.93e-13 (GSE95849), confirming the robustness of the overlap (S9 Table).

To evaluate whether MMP9 and HPSE are consistently prioritized for both anesthetics, we performed parallel analyses separately for sevoflurane and propofol. For sevoflurane, CTD targets (n = 516) and PharmMapper targets (n = 93) were filtered separately, yielding 28 candidate genes from CTD and 6 from PharmMapper(S7a-b Fig). For propofol, CTD targets (n = 472) and PharmMapper targets (n = 137) yielded 7 candidate genes from CTD and 12 from PharmMapper (S7c-d Fig). The combined candidate genes were 33 for sevoflurane and 18 for propofol. The overlap between the two anesthetics revealed 9 common candidate genes, including MMP9 and HPSE, while propofol had 10 specific genes and sevoflurane had 24 specific genes (S7e Fig). MMP9 and HPSE were identified in both anesthetic-specific analyses, supporting their roles as shared molecular targets.

### 3.5. Biological mechanisms of the core targets in type 2 diabetes mellitus

**Fig 6:**
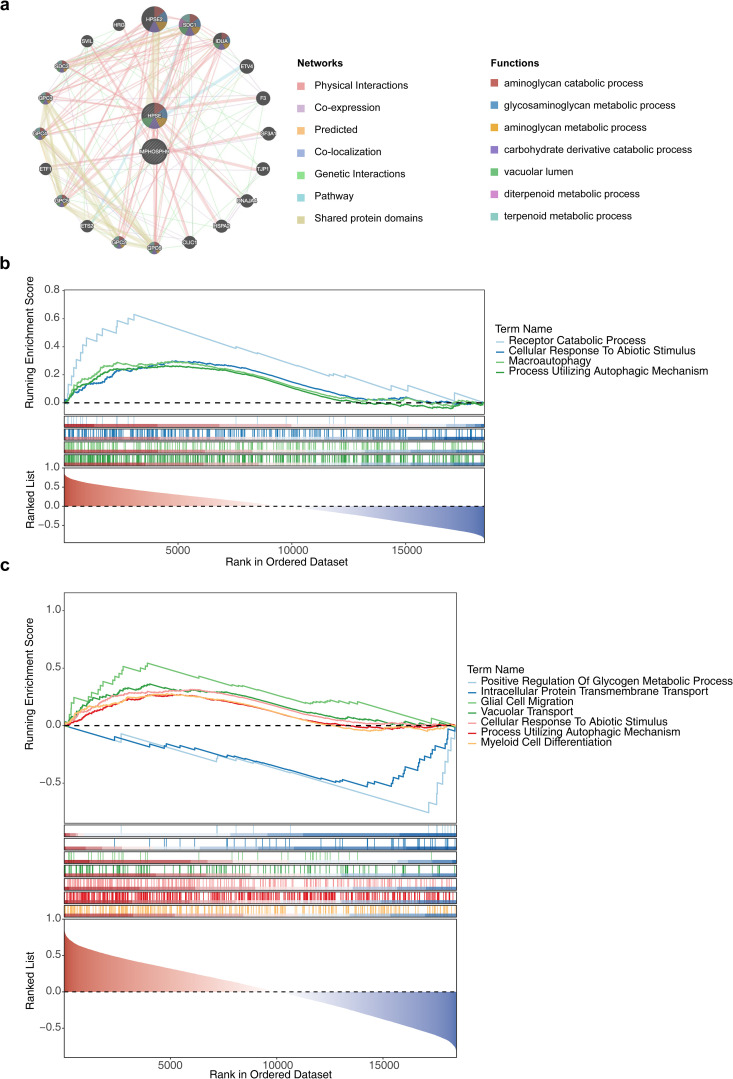
Construction of the co-expression network and gene set enrichment analysis of core targets. **(a)** Co-expression network and functional annotation of core targets and related genes. **(b)** Gene Set Enrichment Analysis (GSEA) of the core target matrix metallopeptidase 9 (MMP9) (p < 0.05, FDR < 0.25, |NES| > 1). **(c)** Gene Set Enrichment Analysis (GSEA) of the core target HPSE (p < 0.05, FDR < 0.25, |NES| > 1).

To further characterize interactions among core targets, the GeneMANIA database was used to construct a co-expression network and explore associated biological functions. The resulting network contained 22 genes, including two core targets and 20 functionally related genes: HPSE2, SDC1, IDUA, ETV4, F3, SF3A1, TJP1, DNAJA4, HSPA2, CLIC1, GPC6, GPC2, ETS2, GPC5, ETF1, GPC4, GPC3, SDC3, SVIL, and HRG. Functional annotation indicated that these genes were primarily involved in biological processes such as “aminoglycan catabolic process” and “glycosaminoglycan (GAG) metabolic process” (Fig 6a).

GSEA was performed to identify coordinated functional alterations associated with core targets. In T2DM, MMP9 was significantly enriched in four pathways, including “receptor catabolic process”, “cellular response to abiotic stimulus”, “macroautophagy”, and “process utilizing autophagic mechanism” (p < 0.05, Fig 6b, S10 Table). HPSE was enriched in seven pathways, including “positive regulation of glycogen metabolic process”, “intracellular protein transmembrane transport”, “myeloid cell differentiation”, “vacuolar transport”, “glial cell migration”, “process utilizing autophagic mechanism”, and “cellular response to abiotic stimulus” (p < 0.05, Fig 6c, S11 Table).

### 3.6. Differential immune cell infiltration between type 2 diabetes mellitus and control groups

**Fig 7:**
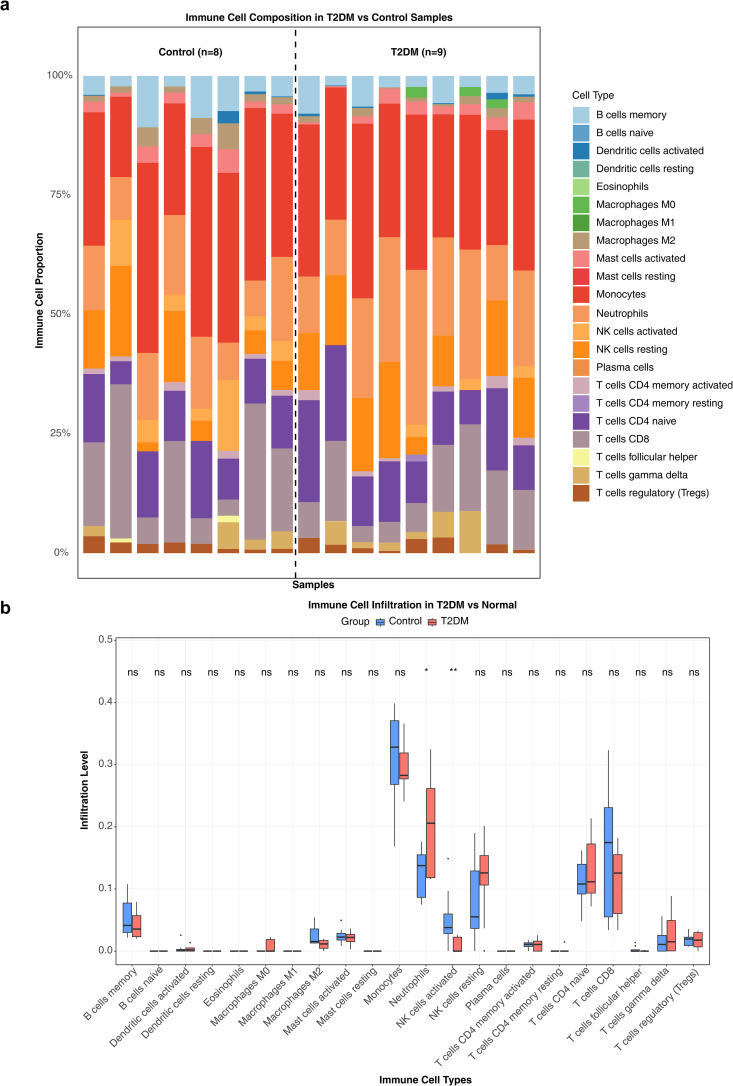
Differential analysis of immune cell infiltration (CIBERSORT and Wilcoxon test). **(a)** Relative proportions of immune cells in the T2DM and control groups. **(b)** Box plots showing differences in immune cell infiltration between groups (*p < 0.05, **p < 0.01, ns indicates no significant difference).

To further characterize the immune microenvironment in T2DM, immune cell infiltration analysis was performed using the GSE21321 dataset to delineate immune cell interactions. The CIBERSORT algorithm was applied to estimate the relative abundance of 22 immune cell types (Fig 7a). Significant differences in immune infiltration were observed between the two groups. Compared to controls, the T2DM group showed an increased proportion of neutrophils and a decreased proportion of activated natural killer (NK) cells (p < 0.05, Fig 7b).

As a supplementary analysis to address multiple comparison concerns, we applied the Benjamini-Hochberg FDR correction to the 22 immune cell type comparisons. After correction, the differences in neutrophils and activated NK cells did not remain statistically significant (adjusted p > 0.05) (S8a-b Fig). These results were presented for transparency, and the original findings should be interpreted with appropriate caution.

### 3.7. Molecular regulatory network of core targets in type 2 diabetes mellitus

**Fig 8:**
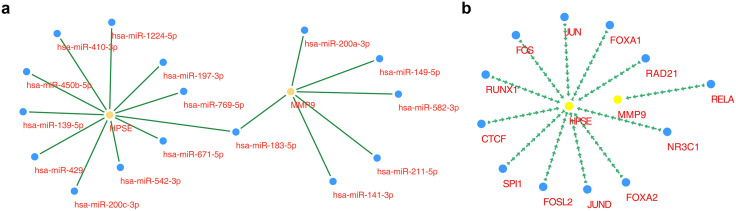
Molecular regulatory networks of core targets. (**a**) miRNA-mRNA regulatory network. Yellow nodes represent core targets, and blue nodes represent corresponding miRNAs. **(b)** Transcription factor (TF)-mRNA regulatory network. Yellow nodes represent core targets, and blue nodes represent corresponding TFs.

Given the critical roles of MMP9 and HPSE, potential regulatory mechanisms were further investigated by predicting associated miRNAs and TFs to construct a multilayer regulatory network. Six miRNAs were predicted to target MMP9, whereas 11 miRNAs were predicted to interact with HPSE. Notable interactions included HPSE-hsa-miR-183-5p and MMP9-hsa-miR-183-5p (Fig 8a). The TF-mRNA network identified 11 TFs targeting HPSE (CTCF, FOS, FOSL2, FOXA1, FOXA2, JUN, JUND, NR3C1, RAD21, RUNX1, and SPI1) and one TF (RELA) targeting MMP9 (Fig 8b). These regulatory networks provide insight into the upstream control mechanisms of MMP9 and HPSE in T2DM.

### 3.8. Molecular docking for sevoflurane and propofol and core targets of type 2 diabetes mellitus

**Fig 9:**
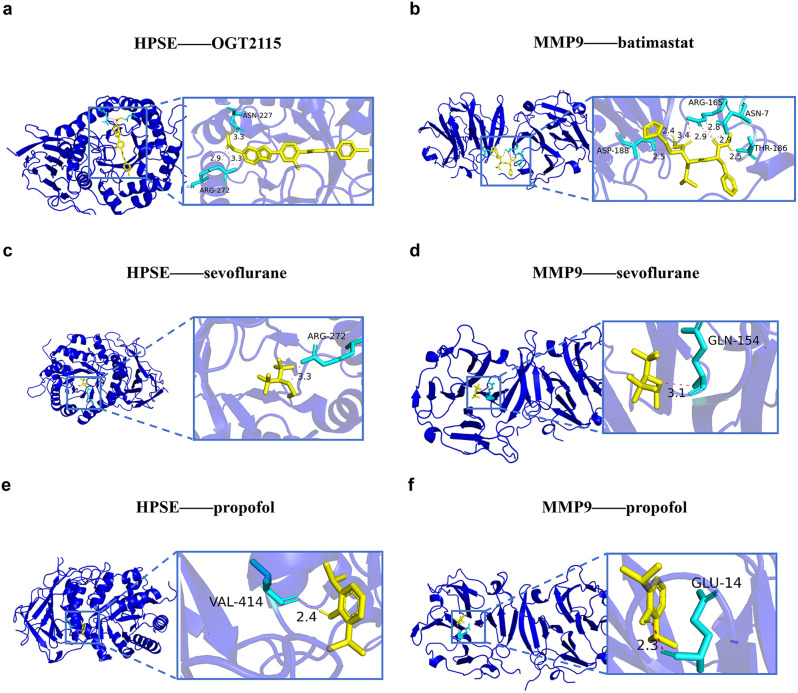
Molecular docking of sevoflurane and propofol with core T2DM targets. **(a)** Docking of the HPSE inhibitor OGT2115 with HPSE. **(b)** Docking of the MMP9 inhibitor Batimastat with MMP9. **(c)** Three-dimensional docking structure of sevoflurane with HPSE (ARG-272). **(d)** Three-dimensional docking structure of sevoflurane with MMP9 (GLN-154). **(e)** Three-dimensional docking structure of propofol with HPSE (VAL-414). **(f)** Three-dimensional docking structure of propofol with MMP9 (GLU-14). Light green ribbon-like structures represent protein receptors (core targets), and stick-like structures represent ligands (sevoflurane and propofol).

The potential binding affinities of sevoflurane and propofol to the two core targets were preliminarily evaluated by molecular docking. Binding energies for the positive control inhibitors Batimastat (MMP9 inhibitor) and OGT2115 (HPSE inhibitor) were −6.5 kcal/mol and −9.3 kcal/mol, respectively (Fig 9a-b). Sevoflurane showed potential binding to HPSE with an energy of −5.1 kcal/mol, involving interactions with residues such as GLN-270 (Fig 9c), and to MMP9 with an energy of −6.0 kcal/mol, interacting with residues including ARG-681 and TYR-672 (Fig 9d). Propofol exhibited potential binding to HPSE with an energy of −5.9 kcal/mol, involving ARG-432 (Fig 9e), and to MMP9 with an energy of −7.1 kcal/mol, involving HIS-401 (Fig 9f).

## 4. Discussion

The incidence of T2DM has increased steadily, highlighting the clinical importance of appropriate anesthetic selection and management during the perioperative period. Previous studies have reported no significant differences in preoperative blood glucose levels between propofol and sevoflurane groups; however, postoperative glucose levels increased in both groups, with significantly lower levels observed in the propofol group, indicating more favorable effects on glucose metabolism and attenuation of surgical stress responses [19]. Through integrated network toxicology and bioinformatics analyses, two core targets, MMP9 and HPSE, were identified, and their potential involvement in the molecular mechanisms associated with sevoflurane and propofol in T2DM was further investigated. Functional attributes, immune infiltration patterns, and upstream regulatory features of these targets were systematically characterized. Molecular docking analyses indicated that the binding affinities of sevoflurane and propofol to these core targets may be linked to diabetes-related molecular pathways. These findings provide preliminary mechanistic insights into the associations between sevoflurane, propofol, and T2DM progression, and highlight candidate molecular targets for further validation and potential therapeutic exploration.

In the T2DM dataset, MMP9 and HPSE were identified as core targets associated with sevoflurane and propofol through integrated multi-method analysis. Matrix metalloproteinase-9 (MMP-9) is a 92 kDa type IV collagenase belonging to the endopeptidase family that mediates extracellular matrix (ECM) degradation, tissue remodeling, receptor shedding, and the regulation of multiple signaling molecules. Previous studies have demonstrated interactions between MMP-9 and integrins, with integrin gene alterations observed in obese individuals and linked to abnormal ECM remodeling. These findings suggest that obesity is associated with increased MMP expression in adipose tissue, with both MMP-2 and MMP-9 contributing to ECM remodeling processes [38]. Neutrophil-driven inflammatory responses and pro-inflammatory cytokines can further enhance MMP-9 activity, promoting microvascular injury and supporting its role as a biomarker of endothelial damage [39]. Clinical evidence indicates that circulating levels of MMP-2 and MMP-9 are significantly elevated in T2DM compared to controls, consistent with the present findings. By modulating cell adhesion, migration, and differentiation in adipose tissue, MMP accumulation exacerbates IR and contributes to impaired glycemic control in T2DM [40]. HPSE is the only mammalian enzyme capable of degrading negatively charged heparan sulfate (HS), and loss of HS within the glomerular basement membrane (GBM) is closely associated with the development of proteinuria [41]. HPSE has been shown to play a central role in the pathogenesis of proteinuria and progressive renal injury in experimental models of glomerulonephritis and diabetic nephropathy [42]. In multiple renal disease models, including diabetic nephropathy, anti-GBM disease, and passive Heymann nephritis, pharmacological inhibition of HPSE reduces proteinuria and improves renal function. Elevated HPSE activity has been documented in T2DM and is strongly implicated in the progression of diabetic nephropathy, in agreement with the current results [43]. Although direct evidence linking anesthetic exposure to the regulation of these targets in T2DM remains limited, previous studies have shown that sevoflurane preconditioning attenuates neurological injury by suppressing microglial MMP9 expression in spinal cord ischemia-reperfusion models [44]. Propofol has been reported to inhibit TNF-α-induced MMP-9 expression in human brain microvascular endothelial cells through suppression of the Ca² ⁺ /CaMK II/ERK/NF-κB signaling pathway [45]. Moreover, HPSE expression is regulated by the receptor for advanced glycation end products (RAGE) *via* NF-κB signaling [46], a pathway also recognized as a key anti-inflammatory mechanism underlying the pharmacological effects of propofol [47]. Thus, sevoflurane and propofol may modulate MMP9 and HPSE expression through NF-κB and related signaling pathways, potentially exacerbating the obesity-induced pro-inflammatory state in patients with T2DM. Further experimental studies are needed to determine whether these anesthetics directly interact with these targets to influence T2DM progression. These findings identify MMP9 and HPSE as key molecular correlates linking sevoflurane and propofol exposure to the pathological features of T2DM and provide a theoretical basis for the development of targeted therapeutic strategies.

Functional enrichment analysis was performed to elucidate the potential biological functions of the core targets. GAGs are linear polysaccharides widely distributed in the ECM and on cell surfaces, primarily including chondroitin sulfate, dermatan sulfate, HS, and hyaluronic acid [48]. Under pathological conditions, GAGs such as chondroitin sulfate and hyaluronic acid are degraded by inflammation-associated enzymes, including hyaluronidase, into low-molecular-weight fragments that function as damage-associated molecular patterns (DAMPs), activating TLR-mediated inflammatory signaling and promoting the release of proinflammatory cytokines such as tumor necrosis factor-α (TNF-α) and interleukin-6 (IL-6). Concurrently, dysregulated GAG metabolism disrupts ECM integrity, impairs tissue barrier function, enhances immune cell infiltration, and exacerbates inflammatory responses [49]. Macroautophagy represents a critical catabolic process responsible for eliminating aberrant intracellular components, maintaining cellular homeostasis, and supplying nutrients under nutrient-deprived conditions. Although stress-induced autophagy initially promotes cell survival, excessive or sustained activation may lead to autophagic cell death. Accordingly, early-stage autophagy supports cellular viability, whereas prolonged or excessive autophagy contributes to programmed cell death and facilitates T2DM progression. Under metabolic stress and endoplasmic reticulum stress-induced IR, autophagy may exert deleterious effects by promoting pancreatic β-cell apoptosis [50]. Accumulating evidence indicates that excessive autophagy activation is closely associated with pancreatic β-cell apoptosis and autophagic cell death [51]. Endoplasmic reticulum stress-mediated IR can further drive autophagy overactivation, a mechanism closely implicated in T2DM pathogenesis [52]. Myeloid cell differentiation is a key process, as abnormal differentiation of myeloid cells—such as monocytes, macrophages, and dendritic cells—can disrupt metabolic balance by regulating immune-inflammatory responses. In turn, the metabolic environment of T2DM can exert a negative feedback on myeloid cell differentiation, creating a vicious cycle. Among the key regulatory factors, the trigger receptor expressed on myeloid cells 2 (TREM2) is a promising immune-metabolic regulator. Primarily expressed on myeloid cells like macrophages, TREM2 promotes the formation of anti-inflammatory macrophage phenotypes and enhances lipid clearance by modulating immune and metabolic responses, thereby mitigating metabolic dysfunction associated with T2DM [53]. Bioinformatic analysis predicted that pathways enriched by MMP9 and HPSE, targets of sevoflurane and propofol, including “utilization of autophagic mechanisms,” intersect with autophagy-related pathological processes. These results suggest that both agents may modulate the autophagy-apoptosis imbalance in T2DM by targeting critical nodes within these pathways, although further experimental validation is required.

To further investigate the immune microenvironment in T2DM, immune infiltration analysis was performed. Before multiple-testing correction, we observed potential trends suggesting differences in neutrophils and activated NK cells between T2DM and control groups. However, after Benjamini-Hochberg FDR correction, no statistically significant differences were detected. These findings should therefore be interpreted with caution and considered hypothesis-generating. Neutrophil extracellular traps (NETs)—fibrous networks composed of DNA fragments, histones, myeloperoxidase (MPO), and neutrophil elastase (NE)—represent a critical facet of neutrophil function [54]. NETs contribute to a range of biological processes, including antimicrobial defense, modulation of inflammation, tissue remodeling, regulation of endothelial cell activity, and thrombogenesis, all of which are potentially linked to the pathogenesis and complications of diabetes. Elevated neutrophil infiltration and increased NET-associated products have been observed in the pancreatic tissue of patients with T2DM [55]. NK cells influence T2DM pathophysiology primarily through modulation of systemic inflammation [56]. Evidence suggests that NK cells are involved in obesity-induced inflammatory responses and the development of IR [57]. A progressive decline in NK cell activity correlates with rising blood glucose levels in T2DM, contributing to immune dysregulation and systemic homeostatic imbalance [58]. Notably, accumulating evidence has reported that sevoflurane and propofol modulate immune cell functions through various signaling pathways, including the NF-κB and MAPK cascades. Specifically, propofol has been validated to inhibit NF-κB activation [59], which may attenuate obesity-induced pro-inflammatory signals. Meanwhile, sevoflurane is closely associated with neutrophil regulation [60]. The potential interactions between these anesthetics and obesity-related immune dysregulation in T2DM warrant further in-depth investigation.Clarifying the functional alterations of NK cells, especially their involvement in inflammatory and insulin-resistant states, may offer novel insights into obesity-associated T2DM.

Integrative analysis of molecular regulatory networks identified hsa-miR-183-5p and other miRNAs as potential modulators in T2DM, meriting further investigation. The network analysis emphasizes the regulatory roles of TFs such as FOXA1 and FOXA2 in mediating the effects of sevoflurane and propofol on T2DM, highlighting their relevance in disease-related pathways. hsa-miR-183-5p, a member of the miR-183 family, is expressed in various tissues, including the pancreas, adipose tissue, liver, and muscle. Its dysregulation has been implicated in metabolic processes, inflammatory responses, and apoptosis. Upregulation of the miR-183 cluster in Th17 lymphocytes enhances production of pathogenic cytokines *via* FOXO1 inhibition, implicating the cluster in autoimmune pathogenesis [61]. FOXA1 and FOXA2, key members of the forkhead box (FOXA) TF family, function as critical activators during the development of endoderm-derived organs such as the liver and pancreas [62]. In mature murine β-cells, simultaneous deletion of FOXA1 and FOXA2 results in more profound disruption of glucose homeostasis and insulin secretion than deletion of FOXA2 alone, highlighting their synergistic role in maintaining β-cell identity and function [63]. Given that impaired β-cell functionality is central to T2DM pathophysiology, dysfunction of FOXA1 and FOXA2 may contribute to disease progression through β-cell impairment. These regulatory elements—hsa-miR-183-5p, FOXA1, and FOXA2—are intricately linked to the molecular underpinnings of T2DM and represent potential mechanistic contributors to its development.

Although the roles of MMP9 and HPSE in T2DM and its complications, including nephropathy and cardiovascular disease, are well established [64,65], their potential involvement in mediating the effects of commonly used anesthetics such as sevoflurane and propofol remains insufficiently characterized. Integrated network toxicology and bioinformatics analyses identified MMP9 and HPSE as potential core molecular intermediaries linking sevoflurane and propofol exposure to T2DM progression. These findings provide a new perspective on the metabolic implications of anesthetic agents and highlight the clinical importance of individualized anesthetic strategies in patients with T2DM. Several limitations should be acknowledged. First, the small sample size (n = 17) limits statistical power and model stability. Although nested cross-validation supported the identification of MMP9 and HPSE, their selection frequencies were moderate (25.7% for MMP9, 35.4% for HPSE), indicating a need for validation in larger cohorts. Second, permutation tests showed that the overall overlap between DEGs, anesthetic targets, and T2DM targets did not reach statistical significance (p = 0.244). Therefore, our findings should be considered hypothesis-generating rather than confirmatory. Third, the molecular docking results, including the predicted binding energy between sevoflurane and HPSE (−5.1 kcal/mol), indicate only theoretical binding potential. Docking methods typically neglect solvent effects and protein conformational dynamics, which may affect predictive accuracy [66]. These findings should therefore be considered hypothesis-generating, and the actual binding affinity and functional relevance require validation using approaches such as surface plasmon resonance, isothermal titration calorimetry, or cell-based functional assays based on native protein structures and validated active sites. Fourth, the transcriptomic datasets used in this analysis lack direct records of anesthetic exposure, precluding causal inference between drug effects and disease phenotypes; accordingly, the conclusions remain theoretical. Moreover, the identification of MMP9 and HPSE was based on public database predictions, and their functional roles as anesthetic-responsive therapeutic targets in T2DM have not yet been experimentally confirmed.

Future research should include multicenter, large-scale prospective clinical studies with systematic collection of peripheral blood samples from patients with T2DM and detailed documentation of anesthetic exposure parameters, including dosage and duration of sevoflurane and propofol administration. Rigorous statistical analyses with appropriate correction for multiple comparisons will be required to validate the robustness and population generalizability of the identified targets and immune signatures. Complementary experimental studies should establish *in vitro* cellular models, such as pancreatic β-cells and hepatocytes, and *in vivo* T2DM animal models. Controlled exposure to sevoflurane or propofol, combined with gene knockout or overexpression strategies and molecular and histopathological analyses, will be essential to determine whether metabolic effects are mediated through MMP9 and HPSE, thereby clarifying the underlying mechanisms and providing experimental support for optimized clinical anesthetic management.

## 5. Conclusions

Network toxicology and bioinformatics analyses identified MMP9 and HPSE as core targets associated with T2DM in the context of sevoflurane and propofol exposure. Functional enrichment indicated that these targets participate in autophagy-related pathways, receptor metabolic processes, and immune regulation. Molecular docking suggested potential binding interactions between sevoflurane or propofol and the core targets MMP9 and HPSE; however, these predictions were based on modeled protein structures, and the estimated binding energies may differ under physiological conditions, requiring experimental validation.

Regulatory network analysis further indicated that specific miRNAs (e.g., hsa-miR-183-5p) and TFs (e.g., FOXA1 and RELA) may function as key upstream regulators mediating anesthetic-associated effects on T2DM-related pathways. These findings provide a preliminary molecular framework linking anesthetic agents to T2DM, identify potential targets for perioperative risk assessment and management, and highlight the importance of individualized anesthetic strategies. Given the reliance on bioinformatic predictions and the limited sample size, future studies incorporating large-scale clinical datasets and experimental validation are necessary to confirm the robustness and biological relevance of these results.

## Supporting information

S1 Fig Two-dimensional molecular structures of sevoflurane (a) and propofol (b). (TIF)

S2 Fig Differentially expressed genes (DEGs) identified using Benjamini-Hochberg FDR correction. (a) Volcano plot of DEGs identified with adjusted p < 0.05 and |log_2_FC| > 0.5, showing only 13 significant DEGs (6 upregulated, 7 downregulated). (b) Heatmap of the 13 FDR-corrected DEGs showing expression patterns between T2DM and control groups.
(TIF)

S3 Fig Permutation test results for the overlap between DEGs, anesthetic targets, and T2DM targets. (a) Histogram and density curve showing the distribution of random overlap counts from 10,000 permutations at FC = 0.50. The red dashed line indicates the observed overlap (43 candidate targets). (b) Frequency distribution of random overlap counts with observed overlap. (c) Fold enrichment across six FC thresholds (0.25, 0.50, 0.75, 1.00, 1.25, 1.50), ranging from 0.8× to 1.1 × , none reaching statistical significance.
(TIF)

S4 Fig Machine learning model validation using nested cross-validation and bootstrap resampling. (a) Distribution of AUC values from 100 repeats of 5-fold nested cross-validation (500 total evaluations). The red dashed line indicates the mean AUC (0.942), and the dashed lines indicate the 95% confidence interval. (b) Boxplot showing six performance metrics (AUC, Accuracy, Sensitivity, Specificity, PPV, NPV) from 100 repeats of nested cross-validation. (c) Distribution of out-of-bag (OOB) AUC values from 1,000 bootstrap iterations. The blue dashed line indicates the mean OOB AUC (0.911). (d) Boxplot showing six performance metrics from 1,000 bootstrap OOB evaluations. (e) Gene selection frequency from 1,000 bootstrap iterations, showing the percentage of iterations in which each gene was selected by the LASSO model. HPSE and MMP9 were selected in 35.4% and 25.7% of iterations, respectively.
(TIF)

S5 Fig Sensitivity analysis across fold-change thresholds for core target retention. (a) Number of DEGs identified at each FC threshold (0.25, 0.50, 0.75, 1.00, 1.25, 1.50), decreasing from 4,034 at FC = 0.25 to 1,811 at FC = 1.50. (b) Number of candidate targets (three-way intersection) at each FC threshold, decreasing from 47 at FC = 0.25 to 15 at FC = 1.50. (c) Retention status heatmap for MMP9 and HPSE across FC thresholds. Both genes were retained at FC = 0.25–0.75; at least one was retained at FC = 1.00–1.25; both were lost only at FC = 1.50, indicating robust identification under moderate threshold selections.
(TIF)

S6 Fig Context-aware filtering of anesthetic targets. (a) Fit score distribution for PharmMapper-predicted targets of propofol. The red dashed line indicates the top 20% threshold (Fit score ≈ 6.12). (b) Fit score distribution for PharmMapper-predicted targets of sevoflurane. The red dashed line indicates the top 20% threshold (Fit score ≈ 5.92). (c) Venn diagram showing the three-way intersection (DEGs ∩ T2DM targets ∩ filtered anesthetic targets) after applying context-aware filtering. The candidate target set was reduced from 43 to 33, and MMP9 and HPSE were retained.
(TIF)

S7 Fig Drug-specific analysis for sevoflurane and propofol. (a) Venn diagram for sevoflurane CTD targets intersecting with T2DM targets and DEGs, yielding 28 candidate genes. (b) Venn diagram for sevoflurane PharmMapper targets intersecting with T2DM targets and DEGs, yielding 6 candidate genes. (c) Venn diagram for propofol CTD targets intersecting with T2DM targets and DEGs, yielding 7 candidate genes. (d) Venn diagram for propofol PharmMapper targets intersecting with T2DM targets and DEGs, yielding 12 candidate genes. (e) Venn diagram showing the overlap of combined candidate genes between sevoflurane (33 genes) and propofol (18 genes). Nine common candidate genes were identified, including MMP9 and HPSE.
(TIF)

S8 Fig Immune infiltration analysis with Benjamini-Hochberg FDR correction. (a) Stacked bar plot showing relative proportions of 22 immune cell types in each sample of the GSE21321 dataset (T2DM vs. control). (b) Boxplots comparing immune cell infiltration between T2DM and control groups after FDR correction. No immune cell type showed statistically significant differences after correction (adjusted p > 0.05).
(TIF)

S1 Table Type 2 diabetes mellitus (T2DM)-related targets. (XLSX)

S2 Table Toxicity-related targets associated with sevoflurane and propofol. (XLSX)

S3 Table Predicted toxicity profiles of sevoflurane and propofol. (XLSX)

S4 Table Differentially expressed genes (DEGs) between the type 2 diabetes mellitus (T2DM) and control groups. (XLSX)

S5 Table Candidate target genes. (XLSX)

S6 Table Gene Ontology (GO) enrichment results of candidate targets (1,001 terms). (XLSX)

S7 Table Kyoto encyclopedia of genes and genomes (KEGG) pathway enrichment results of candidate targets (87 pathways). (XLSX)

S8 Table Candidate target genes after context-aware filtering (n = 33). (XLSX)

S9 Table Hypergeometric test p-values using different background gene sets. (XLSX)

S10 Table Gene set enrichment analysis results for the core target MMP9. (XLSX)

S11 Table Gene set enrichment analysis results for the core target HPSE. (XLSX)
