# Supplementary figures and images for: Network toxicology and bioinformatics analysis predict potential molecular targets and mechanisms by which sevoflurane and propofol influence type 2 diabetes mellitus
Source: PLoS One. 2026 May 18;21(5):e0349565. doi: 10.1371/journal.pone.0349565 (PMC13183223; doi:10.1371/journal.pone.0349565)

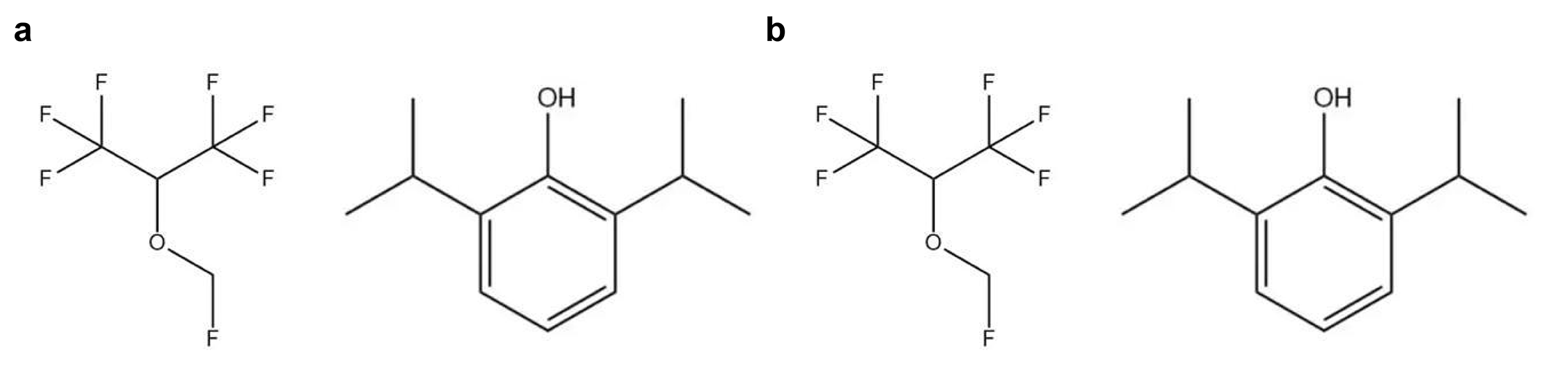

Supplement: S1 Fig — (TIF) [file pone.0349565.s001.tif]

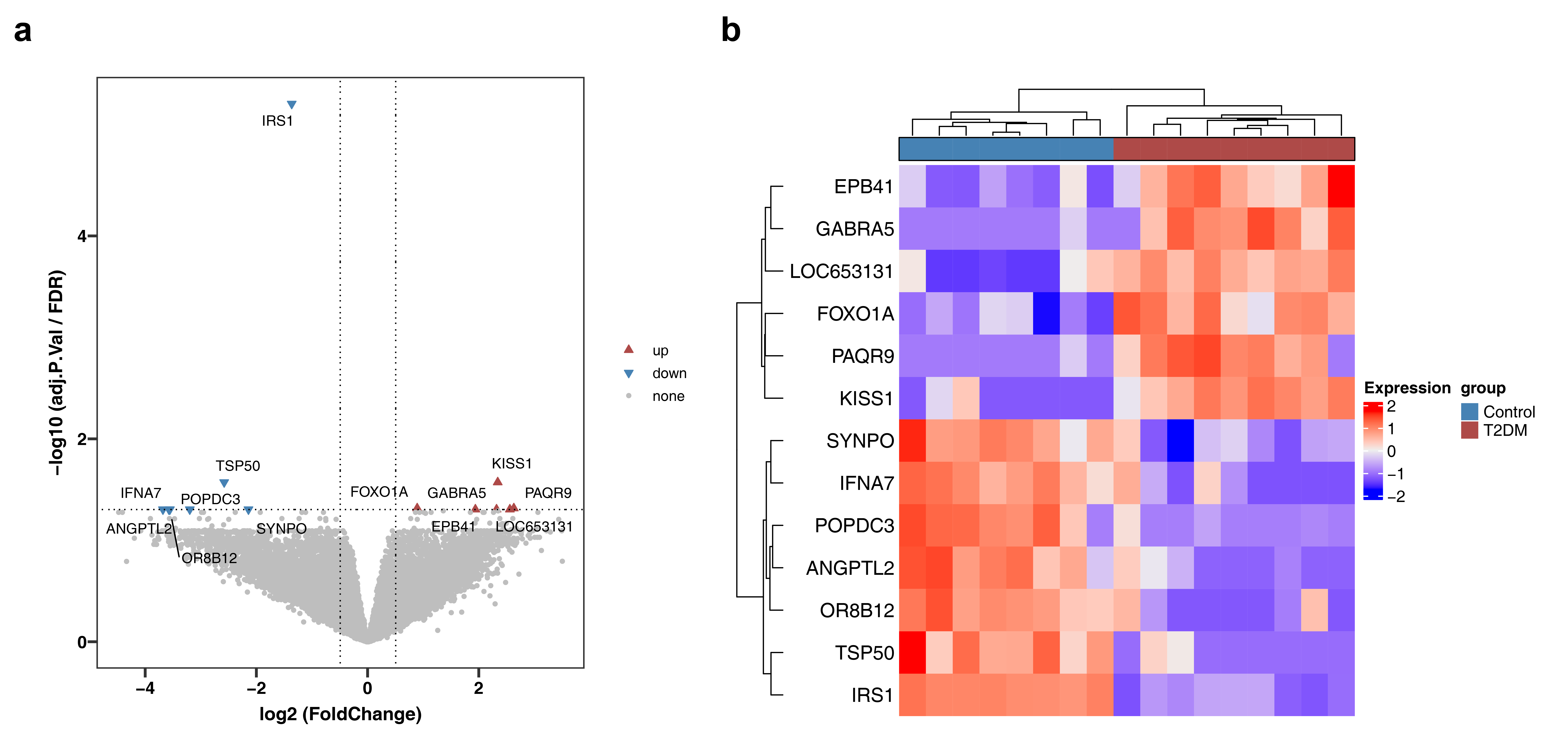

Supplement: S2 Fig — (a) Volcano plot of DEGs identified with adjusted p < 0.05 and |log2FC| > 0.5, showing only 13 significant DEGs (6 upregulated, 7 downregulated). (b) Heatmap of the 13 FDR-corrected DEGs showing expression patterns between T2DM and control groups. (TIF) [file pone.0349565.s002.tif]

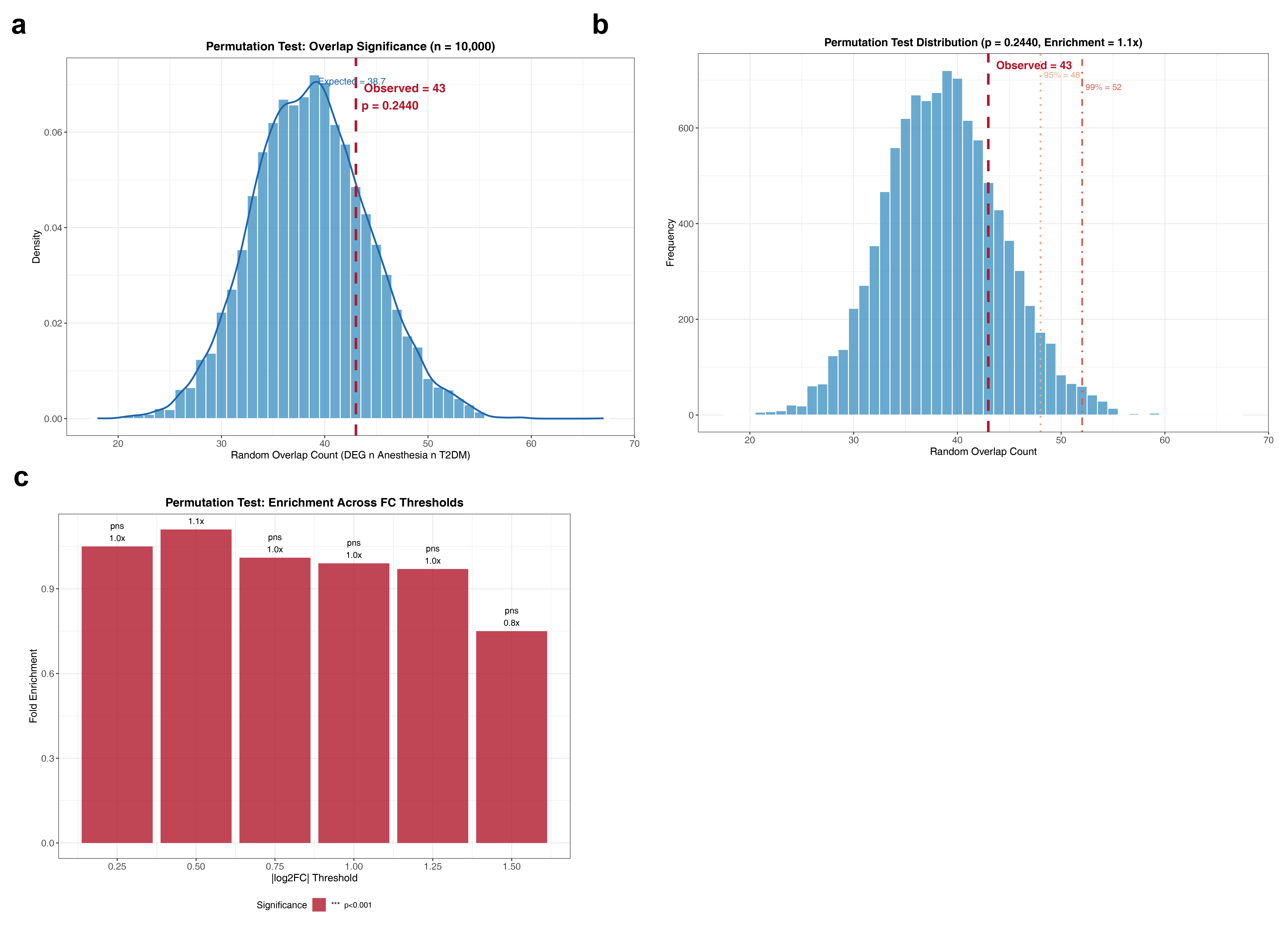

Supplement: S3 Fig — (a) Histogram and density curve showing the distribution of random overlap counts from 10,000 permutations at FC = 0.50. The red dashed line indicates the observed overlap (43 candidate targets). (b) Frequency distribution of random overlap counts with observed overlap. (c) Fold enrichment across six FC thresholds (0.25, 0.50, 0.75, 1.00, 1.25, 1.50), ranging from 0.8× to 1.1 × , none reaching statistical significance. (TIF) [file pone.0349565.s003.tif]

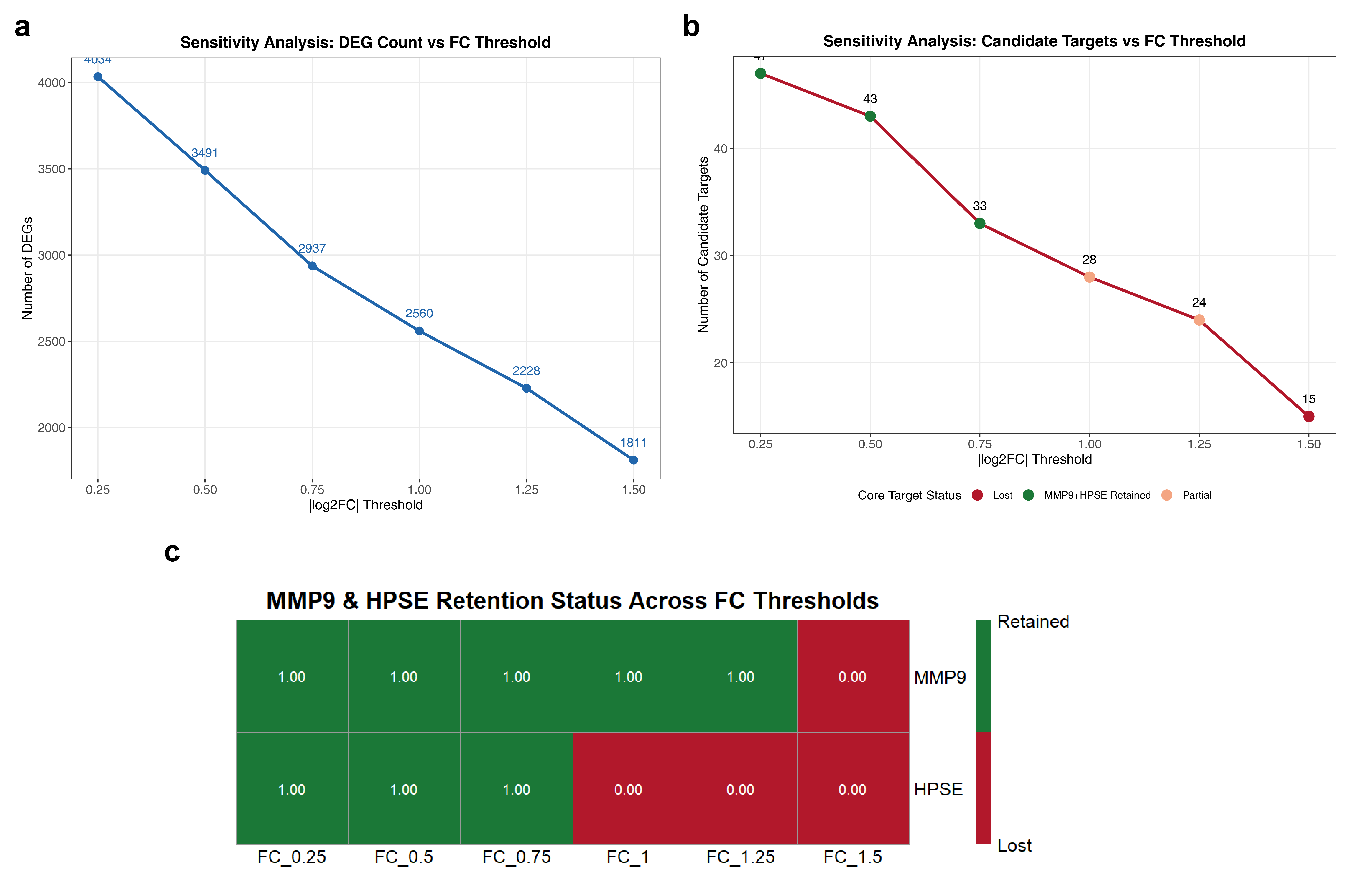

Supplement: S5 Fig — (a) Number of DEGs identified at each FC threshold (0.25, 0.50, 0.75, 1.00, 1.25, 1.50), decreasing from 4,034 at FC = 0.25 to 1,811 at FC = 1.50. (b) Number of candidate targets (three-way intersection) at each FC threshold, decreasing from 47 at FC = 0.25 to 15 at FC = 1.50. (c) Retention status heatmap for MMP9 and HPSE across FC thresholds. Both genes were retained at FC = 0.25–0.75; at least one was retained at FC = 1.00–1.25; both were lost only at FC = 1.50, indicating robust identification under moderate threshold selections. (TIF) [file pone.0349565.s005.tif]

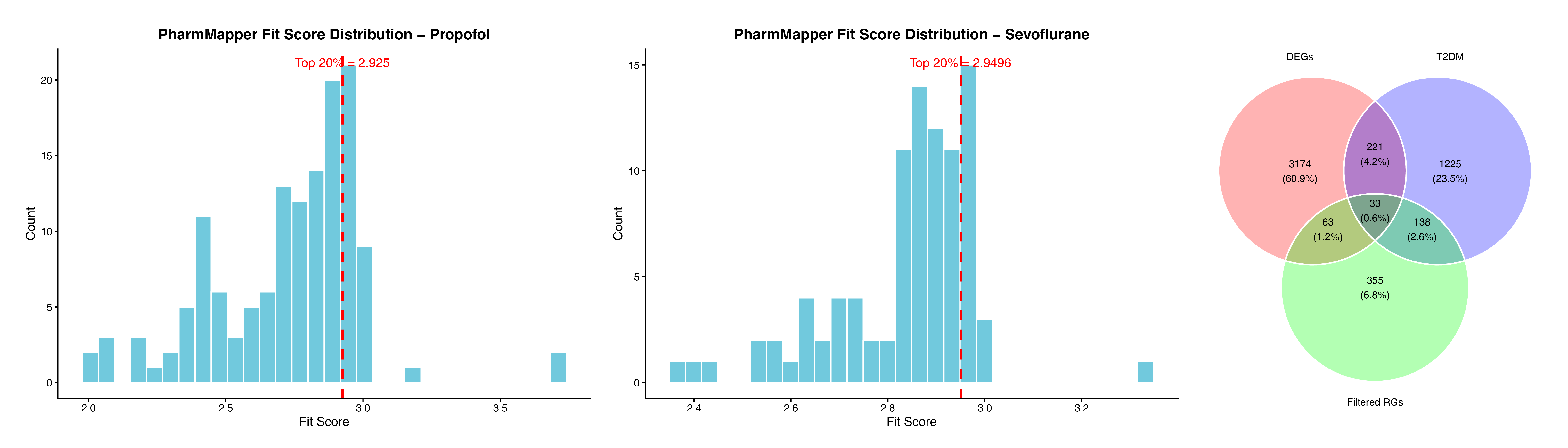

Supplement: S6 Fig — (a) Fit score distribution for PharmMapper-predicted targets of propofol. The red dashed line indicates the top 20% threshold (Fit score ≈ 6.12). (b) Fit score distribution for PharmMapper-predicted targets of sevoflurane. The red dashed line indicates the top 20% threshold (Fit score ≈ 5.92). (c) Venn diagram showing the three-way intersection (DEGs ∩ T2DM targets ∩ filtered anesthetic targets) after applying context-aware filtering. The candidate target set was reduced from 43 to 33, and MMP9 and HPSE were retained. (TIF) [file pone.0349565.s006.tif]

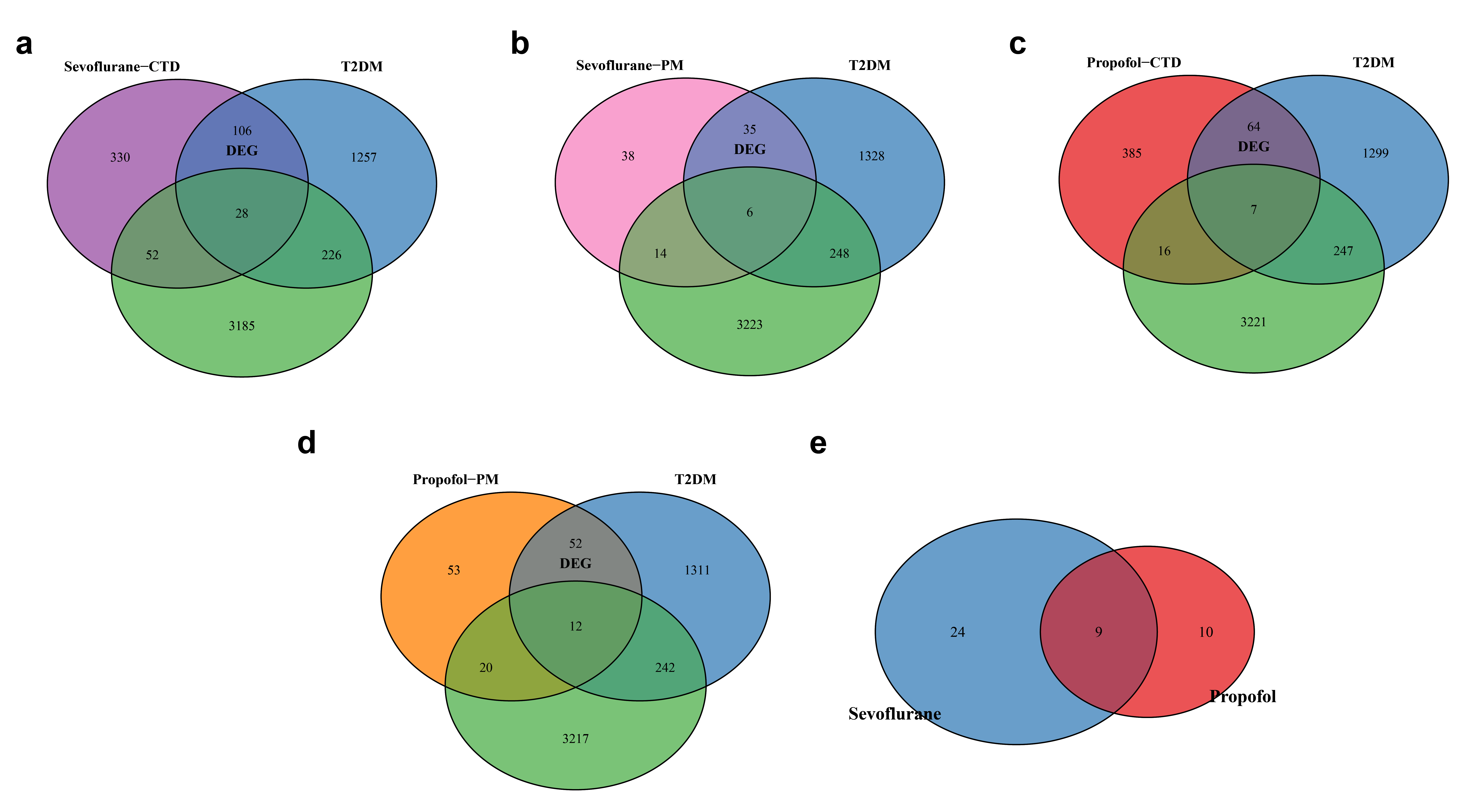

Supplement: S7 Fig — (a) Venn diagram for sevoflurane CTD targets intersecting with T2DM targets and DEGs, yielding 28 candidate genes. (b) Venn diagram for sevoflurane PharmMapper targets intersecting with T2DM targets and DEGs, yielding 6 candidate genes. (c) Venn diagram for propofol CTD targets intersecting with T2DM targets and DEGs, yielding 7 candidate genes. (d) Venn diagram for propofol PharmMapper targets intersecting with T2DM targets and DEGs, yielding 12 candidate genes. (e) Venn diagram showing the overlap of combined candidate genes between sevoflurane (33 genes) and propofol (18 genes). Nine common candidate genes were identified, including MMP9 and HPSE. (TIF) [file pone.0349565.s007.tif]

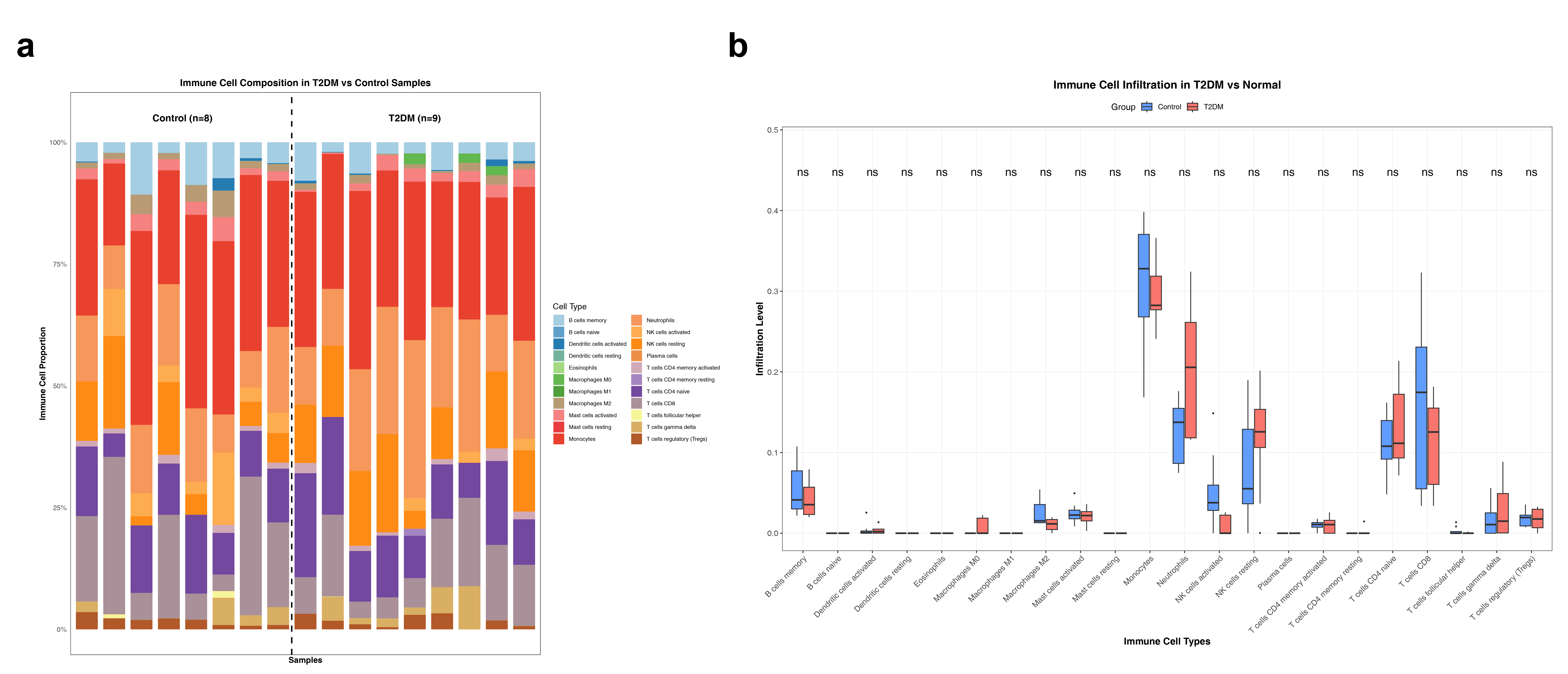

Supplement: S8 Fig — (a) Stacked bar plot showing relative proportions of 22 immune cell types in each sample of the GSE21321 dataset (T2DM vs. control). (b) Boxplots comparing immune cell infiltration between T2DM and control groups after FDR correction. No immune cell type showed statistically significant differences after correction (adjusted p > 0.05). (TIF) [file pone.0349565.s008.tif]
